# Cardiac function and coronary plaque development following masculinizing gender‐affirming hormone therapy: A prospective cohort study

**DOI:** 10.1111/andr.13832

**Published:** 2025-01-13

**Authors:** Laust Frisenberg Buhl, Marianne S. Andersen, Jan Frystyk, Axel Diederichsen, Selma Hasific, Rikke Hjortebjerg, Jordi Sanchez Dahl, Manijeh Noori, Kirstine Nørregaard Hansen, Gitte Maria Jørgensen, Camilla Viola Palm, Tine Taulbjerg Kristensen, Dorte Glintborg, Louise Lehmann Christensen

**Affiliations:** ^1^ Department of Endocrinology Odense University Hospital Odense Denmark; ^2^ Clinical Institute Faculty of Health Sciences University of Southern Denmark Odense Denmark; ^3^ Department of Cardiology Odense University Hospital Odense Denmark; ^4^ STENO Diabetes Center Odense Odense University Hospital Odense Denmark; ^5^ Department of Radiology Odense University Hospital Odense Denmark

**Keywords:** cardiac function, coronary plaque formation, masculinizing gender‐affirming hormone therapy

## Abstract

**Introduction:**

Myocardial dysfunction and the presence of calcified and non‐calcified coronary plaques are predictors of cardiovascular disease. Masculinizing gender‐affirming hormone therapy may increase cardiovascular risk, highlighting the need for prospective studies to evaluate cardiovascular outcomes during gender‐affirming hormone therapy.

**Objectives:**

To evaluate changes in cardiac morphology, systolic and diastolic function, and development of coronary plaques after masculinizing gender‐affirming hormone therapy.

**Methods:**

Prospective study including 47 transmasculine persons (gender‐affirming hormone therapy‐naïve, TransM_TN, *n* = 15 and gender‐affirming hormone therapy‐ongoing, TransM_TO, *n* = 32). Included persons were evaluated at study inclusion and after one year of masculinizing gender‐affirming hormone therapy. At baseline, the median age of TransM_TN was 22 years (interquartile range 19–28 years) and TransM_TO 26 years (interquartile range 24–37 years) with a median gender‐affirming hormone therapy duration of 4 years (interquartile range 2–5 years). Cardiac morphology including left ventricular wall thickness, volume, and mass, as well as left ventricular systolic and diastolic function was evaluated using echocardiography. Coronary artery calcifications and non‐calcified coronary plaque were assessed using coronary computed tomography angiography. Paired and unpaired statistical analyses were performed within and between TransM_TN and TransM_TO groups.

**Results:**

In TransM_TN, diastolic function decreased during follow‐up with decreased septal and lateral left ventricular relaxation (14–11 cm/s, *p* = 0.04 and 18–15 cm/s, *p* = 0.02, respectively). No significant changes were observed in cardiac morphology, systolic function, or formation of coronary artery calcifications and non‐calcified coronary plaque in TransM_TN or TransM_TO groups. At baseline, left ventricular end‐diastolic internal diameter was significantly higher in TransM_TO compared to TransM_TN, 4.6 cm (interquartile range 4.3–5.0 cm) versus 4.4 cm (interquartile range 4.2–4.6 cm), *p* < 0.05. Other baseline cardiac outcomes were comparable between TransM_TN and TransM_TO.

**Conclusion:**

Diastolic function declined after the initiation of masculinizing gender‐affirming hormone therapy and individuals on long‐term masculinizing gender‐affirming hormone therapy had larger left ventricular dimensions compared to individuals before gender‐affirming hormone therapy initiation. Cardiac morphology, systolic function, and coronary plaque formation remained stable during masculinizing gender‐affirming hormone therapy.

## 1 INTRODUCTION

Transgender men are assigned female sex at birth but identify as male. Transgender identity may lead to gender dysphoria and the desire for gender‐affirming hormone therapy (GAHT).^1^ The aim of masculinizing GAHT is to align physical appearance and gender identity ^2^ and improve mental health outcomes. However, GAHT is generally lifelong and the potential adverse effects on cardiac function and cardiovascular risk of masculinizing GAHT are debated. A study involving 1,358 transgender men from the Amsterdam cohort, who underwent masculinizing GAHT for an average duration of eight years, reported a threefold increase in the incidence of myocardial infarctions in transmasculine persons compared to cisgender women,^3^ which was comparable to the cardiac risk for cisgender men.^3^ Additionally, a cross‐sectional study found that transgender men had 2–4 fold higher reported history of myocardial infarction compared to both cisgender men and cisgender women after adjustment for cardiovascular risk factors, but the study included no information on GAHT treatment modality or GAHT duration.^4^ A recent meta‐analysis of 10 studies involving 11,304 transmasculine persons found a higher incidence of myocardial infarction compared to cisgender women, with a pooled relative risk of any cardiovascular disease (CVD) outcome of 1.3 (95% confidence interval, 1.0–1.7).^5^ However, the studies included in the meta‐analysis had limitations, such as cross‐sectional or retrospective designs, and not all transgender men were using GAHT.^5^

There are no prospective studies on the effect of masculinizing GAHT on clinical cardiac outcomes or the timeframe of the development of possible cardiac changes after initiating masculinizing GAHT. Physiologically female and male hearts have comparable sizes before puberty.^6^ Adult female hearts are generally smaller than male hearts,^7^ but the female heart is not simply a smaller version of the male heart. Disproportionate sex differences are observed in ventricular chamber size and wall thickness.^8^ In cisgender women, ejection fraction and ventricular contractility tend to be higher compared to cisgender men, and heart rates are typically higher.^8^ However, there is limited data on the effects of masculinizing GAHT on the female heart, leaving gaps in our understanding of how masculinizing GAHT impacts cardiac structure and function.^8^ The exact mechanism for the development of atherosclerotic disease in transgender men remains a subject of ongoing discussion.^9^, ^10^ Masculinizing GAHT increases triglyceride levels and plasma LDL cholesterol, whereas plasma HDL cholesterol decreases.^11^, ^12^, ^13^ Furthermore, higher blood pressure,^14^, ^15^ and hematocrit levels^16^ during masculinizing GAHT could be risk factors for the development of atherosclerosis,^17^, ^18^, ^19^ and endothelial function was impaired during masculinizing GAHT.^20^ The average age of initiation of masculinizing GAHT is around 22 years ^21^ and at this age, the baseline risk for adverse CVD outcomes is expected to be low.^22^ Masculinizing GAHT will be lifelong in most cases, and the risk of cardiovascular complications could increase over time.^23^ In young hypogonadal cis‐gender men, the risk of CVD was not increased during testosterone replacement therapy.^24^ In contrast, coronary plaque formation increased after only 1 year of testosterone replacement therapy in older cisgender men.^25^

The aim of this study was to assess prospective changes in cardiac ventricular morphology, systolic and diastolic function, coronary artery calcium scores, non‐calcified plaque formation, degree of coronary stenosis, and cardiovascular risk factors during masculinizing GAHT.

## 2 METHODS

### 2.1 Study design, participants, and masculinizing treatment

**FIGURE 1:**
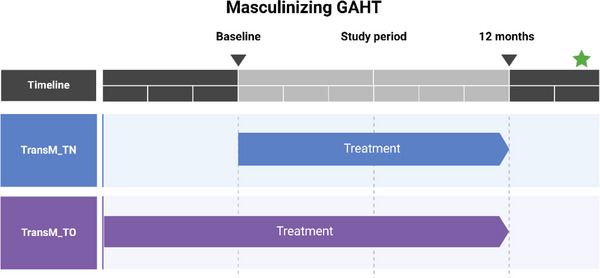
Study Timeline. The figure depicts a study timeline showing the two different groups: TOxM (transgender males on gender‐affirming hormone therapy [GAHT] prior to baseline) and TNxM (transgender males starting GAHT at baseline) during the study period.

Single‐center, prospective study at Odense University Hospital including 47 transgender men (GAHT‐naïve, TransM_TN, *n* = 15 and GAHT‐ongoing, TransM_TO, *n* = 32) (Figure 1).

**Inclusion criteria**: Persons assigned female at birth (+18 years) that either use or want to use testosterone with the purpose of undergoing gender transformation.

**Exclusion criteria**: Psychiatric, mental, or severe somatic diseases that made it impossible for the study person to give informed consent or to comply with the investigatory program.

The study outcomes were evaluated at baseline and repeated after 1 year of masculinizing GAHT prescribed according to the Endocrine Society Clinical Practice Guidelines for Gender Dysphoria^26^ with testosterone as gels or injections. The treatment regimen adhered to international guidelines, targeting serum testosterone levels within the normal range for cisgender men.

Masculinizing GAHT in the TransM_TN group began with transdermal testosterone gel, with a gradual dose escalation from 10 mg to 30–50 mg per day over the first four months. Subsequently, participants chose between continuing with transdermal testosterone gel or switching to intramuscular (IM) testosterone injections (testosterone undecanoate 1000 mg every 12 weeks) based on their preference.

We collected information regarding chronic diseases, medications, alcohol, and tobacco.

All participants provided informed consent and Research Electronic Data Capture (REDCap®) was used for data management.^27^, ^28^

### 2.2 Echocardiography

Echocardiography was performed in accordance with guidelines^29^, ^30^ on Vivid E95 (General Electric Healthcare, Horten, Norway), by experienced physicians. Doppler values were calculated with a horizontal sweep speed of 100 cm/s and a minimum frame rate of 60 fps. We assessed key **morphological parameters**, including intraventricular septum thickness, left ventricular end‐diastolic internal diameter, left ventricular end‐systolic internal diameter, posterior wall diameter, and left atrial volume normalized to body surface area (left atrial volume index). The left ventricular mass index was estimated using Devereux's formula.^31^ To further evaluate left ventricular geometry, relative wall thickness (RWT) was calculated using the formula: RWT = (2 × LV posterior wall thickness)/LV internal diameter in diastole. RWT values greater than 0.42 indicate concentric remodeling or hypertrophy.^31^

**Systolic function** was assessed by calculating left ventricular ejection fraction (LVEF) using the Simpsons biplane method, derived from both apical 4‐ and 2‐chamber views, supplemented by automated analysis (Auto LVEF) performed by the advanced echocardiography system.^32^ Global longitudinal strain, assessed using speckle tracking of 18 myocardial segments, provided a more sensitive marker of systolic function^33^ Left ventricular stroke volume was calculated as the product of the LV outflow area and the LV outflow tract velocity time integral. Cardiac output, representing the total volume of blood the heart pumps per minute, was calculated by multiplying stroke volume by heart rate.

**Diastolic function** was assessed using both septal and lateral early ventricular relaxation velocities (E’) derived from tissue Doppler imaging.^34^

### 2.3 Coronary computed tomography angiography

Coronary computed tomography (CT) angiography was performed using a Siemens Somatom Force CT scanner with participants in the supine position and arms raised above the head. Both non‐contrast and contrast‐enhanced ECG‐gated CT scans were conducted, with the scanning protocol dependent on the heart rate. For patients with a stable heart rate above 60 beats per minute, intravenous β‐blockers were administered to lower the heart rate to below 60 bpm if possible, allowing for a prospectively gated end‐diastolic scan. If the heart rate remained above 70 bpm after β‐blocker pretreatment, or in cases of irregular heart rhythm, a prospective scan 250–400 ms after the QRS complex was performed. In accordance with clinical practice, β‐blockers and sublingual nitrates were administered prior to CT scans only when required to optimize image quality in cases of elevated heart rates or irregular rhythms.

Images were reconstructed with a slice thickness of 0.5 mm and were used to assess coronary artery calcium (CAC) score, calcified/non‐calcified plaque volumes, and coronary stenosis (%) by experienced cardiologists. Non‐contrast CT scans for CAC scoring were analyzed using the Agatston method with Syngo. CT CaScoring software (Siemens Healthcare). Contrast CT scans were visually evaluated for plaque and coronary stenosis. For plaque areas, calcified and non‐calcified plaque quantification was performed by a trained physician using deep learning software (AutoPlaque, Version 3.0; Cedars Sinai Medical Center). Quantitative analysis was conducted on lesions in vessels with a distal normal reference diameter ≥ 2.0 mm. Plaque volume at the per‐patient level was the primary assessment for plaque types. Coronary artery stenosis was visually assessed on a 17‐segment basis using contrast CT scans.^35^

### 2.4 Biochemical analyses

Serum total testosterone (TT) was measured using liquid chromatography‐tandem mass spectrometry (LC‐MS/MS) after an overnight fast between 8:00 and 9:00 AM. Sex hormone‐binding globulin (SHBG) was measured using a Roche assay with a precision range of 1.8% to 4.0%. Free testosterone (FT) levels were calculated assuming a plasma albumin concentration of 43 g/L.^36^

Hematocrit was measured using an automated cell counter (CV < 3 %). Total cholesterol and HDL cholesterol were analyzed by enzymatic colorimetric methods and LDL cholesterol was calculated.^37^

#### 2.4.1 Dual‐energy X‐ray absorptiometry

Lean body mass and whole‐body fat were measured by dual‐energy X‐ray absorptiometry (DXA) (Hologic Discovery) according to standardized protocols.

### 2.5 Physical examination

Height (m), weight (kg), body mass index (BMI, kg/m^2^), waist (cm), heart rate (bpm), and blood pressure (mmHg) were measured.

### 2.6 Sample size and statistical analysis

The primary endpoint was the presence and volumetric assessment of coronary non‐calcified plaques (mm^3^), evaluated using contrast‐enhanced coronary CT angiography.^35^ The sample size calculation for the full study estimated that 100 participants would be needed to detect a 15% change in coronary non‐calcified plaque volume with 80% power and a significance level of α = 0.05. This interim analysis focuses on the echocardiographic findings of the study cohort. At the time of study planning, no prior data were available to enable a power calculation specifically for echocardiographic outcomes.

Continuous variables were presented as median with interquartile range or mean with standard deviation (SD). Categorical variables were shown as frequency (*n*) with corresponding percentages (%). Data distribution was assessed using Q‐Q plots and Shapiro‐Wilk tests. Logarithmic transformation was applied to non‐normal data to enhance suitability for analysis.

Paired t‐tests were used to assess the significance of changes in normally distributed continuous variables, while Wilcoxon signed‐rank tests were used for non‐normally distributed variables. Categorical variables were compared using chi‐squared tests.

Delta (Δ) estimated differences with 95% confidence intervals (CIs) were used to present changes between baseline and follow‐up measurements for each participant with normally distributed Δ values.

Baseline values were compared between the TransM_TN and the TransM_TO group.

All tests were two‐sided, with p < 0.05 considered significant. Sensitivity assessments were conducted to check robustness, including outlier identification and removal. We also performed a sensitivity analysis in which participants with identified risk factors (defined as the use of antidiabetic medication, cholesterol‐lowering drugs, and antihypertensive treatment) were excluded. This analysis aimed to determine whether excluding these participants influenced the observed results and trends in the primary and secondary outcome analyses. Statistical analyses were performed using Stata software version 18 (StataCorp LP).

## 3 RESULTS

The study cohort included 47 transmasculine persons (15 TransM_TN and 32 TransM_TO with a median duration of GAHT prior to enrollment of 4 years, range 2–5 years). The median age (interquartile range) was 22 years ^19^, ^20^, ^21^, ^22^, ^23^, ^24^, ^25^, ^26^, ^27^, ^28^ for the TransM_TN group and 26 years^24^, ^25^, ^26^, ^27^, ^28^, ^29^, ^30^, ^31^, ^32^, ^33^, ^34^, ^35^, ^36^, ^37^ for the TransM_TO group. In the TransM_TN group, all 15 participants used testosterone gel at baseline, decreasing to two participants at the 1‐year follow‐up.

In the TransM_TO group, four participants used testosterone gel both at baseline and at the 1‐year follow‐up.

In the TransM_TN group, no participants were prescribed antidiabetic or cholesterol‐lowering medication, and one participant was prescribed antihypertensive medication at baseline and at follow‐up. In the TransM_TO group, one participant was prescribed antidiabetics, two cholesterol‐lowering drugs, and one antihypertensive medication at baseline and at follow‐up.

In the TransM_TN group, eight individuals never smoked, five were previous smokers, and two were current smokers. In the TransM_TO group, twenty‐two participants never smoked, five were previous smokers, and five were current smokers.

Baseline and 1‐year clinical data are presented in Table 1.

**TABLE 1:** Baseline and 12 months data transgender men, treatment‐naive (*n *= 15) and transgender men, treatment ongoing (*n* = 32).

	Baseline	12 months	Δ Estimated differences (95% CI)	*p*‐value
Age (years) TransM_TN TransM_TO	22 (19;28) 26 (24;37)*	23 (21;29) 27 (25;38)*	1.2 (1.1;1.3) 1.0 (1.0;1.1)	
Weight (kg) TransM_TN TransM_TO	69 (64;78) 67 (61;82)	68 (66;76) 68 (62;84)	−1.4 (−4.0;1.3) 1.1 (−0.5;2.7)	0.29 0.18
BMI (kg/m^2^) TransM_TN TransM_TO	25.6 (22.3;29.2) 24.9 (22.0;28.4)	24.3 (21.6;27.8) 25.1 (22.3; 29.2)	−0.67 (−1.65;0.32) 0.36 (−0.20;0.92)	0.17 0.20
Waist (cm) TransM_TN TransM_TO	83 (81;93) 84 (76;97)	86 (74;88) 85 (78;97)	−3.1 (−6.5;0.2) 1.1 (−0.5;2.6)	0.07 0.162
Lean body mass (kg) TransM_TN TransM_TO	37.0 (36.1;39.1) 42.0 (37.5;49.0)*	40.0 (38.7;42.6) 42.3 (38.2;48.3)	**3.4 (2.8;4.0)** 0.2 (−0.4;0.9)	** < 0.01** 0.462
Whole‐body fat (kg) TransM_TN TransM_TO	25.5 (21.3;34.6) 20.1 (17.1;27.1)	21.4 (19.7;29.6) 21.2 (16.8;29.6)	**−4.2 (−6.8;−1.5)** 0.8 (−0.4;2.1)	** < 0.01** 0.176
Systolic blood pressure (mmHg) TransM_TN TransM_TO	120 (116;124) 130 (121;144)*	121 (116;126) 127 (118;141)	0.2 (−5.3;5.6) −2.2 (−6.5;2.2)	0.95 0.32
Diastolic blood pressure (mmHg) TransM_TN TransM_TO	73 (67;74) 77 (71;84)*	70 (67;83) 75 (70;84)	0.6 (−3.4;4.6) −1.2 (−3.7;1.4)	0.76 0.36
TT (nmol/l) TransM_TN TransM_TO	0.81 (0.78;1.0) 17.7 (12.1;32.7)*	19.5 (9.7;23.2) 17.5 (14.3;22.6)	**18.6 (10.9;26.4)** −2.9 (−8.7;2.9)	** < 0.01** 0.31
FT (nmol/l) TransM_TN TransM_TO	0.02 (0.02;0.02) 0.37 (0.30;0.74)*	0.42 (0.23;0.53) 0.44 (0.34;0.58)	**0.46 (0.23;0.70)** −0.07 (−0.23;0.09)	**0.001** 0.38
Hematocrit (%) TransM_TN TransM_TO	42 (41;43) 46 (45;48)*	46 (43;48) 46 (45;49)	**4.3 (2.7;6.0)** 0.4 (−0.6;2.0)	** < 0.01** 0.40
Creatinine (µmol/L) TransM_TN TransM_TO	62 (56;67) 78 (70;87)*	75 (70;78) 81 (72;90)	**12 (8;16)** 1.4 (−1.3;4.2)	** < 0.01** 0.30
Total cholesterol (mmol/L) TransM_TN TransM_TO	3.9 (3.5;4.8) 4.3 (3.9;4.9)	4.0 (3.7;4.6) 4.3 (3.9;4.9)	0.0 (−0.3;0.3) 0.1 (−0.1;0.3)	0.89 0.54
HDL cholesterol (mmol/L) TransM_TN TransM_TO	1.4 (1.2;1.9) 1.3 (1.1;1.7)	1.2 (1.0;1.5) 1.3 (1.1;1.6)	**−0.2 (−0.4;−0.1)** 0.0 (−0.1;0.1)	** < 0.01** 0.73
LDL cholesterol (mmol/L) TransM_TN TransM_TO	2.0 (1.7;2.9) 2.4 (2.1;3.2)	2.4 (1.6;3.0) 2.6 (2.1;3.0)	0.2 (−0.2;0.5) 0.1 (−0.0;0.3)	0.24 0.11
Triglycerides (mmol/L) TransM_TN TransM_TO	0.8 (0.5;1.1) 0.9 (0.7;1.3)	0.9 (0.6;1.1) 1.0 (0.7;1.4)	0.1 (−0.1;0.2) 0.1 (−0.1;0.2)	0.28 0.49

**Legend** Table 1: Baseline and 12 months data.

Data presented as median (interquartile range), number (*n*), or percentage (%) of persons. Delta (Δ) differences present changes between baseline measurements and after one year of testosterone therapy for biochemical markers with normally distributed delta values. *P*‐values represent paired comparisons of the difference between baseline and 12 months.

Abbreviations: FT: Free testosterone, HbA1c: Glycated hemoglobin, HDL: High‐density lipoprotein, LDL: Low‐density lipoprotein, BMI: Body mass index.; TransM_TN: Transgender men treatment‐naive, TransM_TO: Transgender men treatment ongoing, TT: Total testosterone.

* *p* < 0.05 when comparing values between new transgender males and transgender males already on treatment at baseline or 12 months, respectively.

### 3.1 Baseline compared to 1‐year assessments

In TransM_TN, TT, FT, hematocrit, and lean body mass significantly increased, while HDL cholesterol and whole‐body fat significantly decreased after 1 year. Total Cholesterol, LDL cholesterol, triglycerides, and blood pressure were unchanged.

No prospective significant changes were observed in the TransM_TO group.

### 3.2 Baseline assessments—TransM_TO vs. TransM_TN

TT, FT, hematocrit, lean body mass, and diastolic blood pressure were significantly higher in the TransM_TO group compared to the TransM_TN group at baseline.

### 3.3 Echocardiography

Echocardiographic data at baseline and 1 year are presented in Table 2.

**TABLE 2:** Echocardiographic data transgender men, treatment‐naive (*n* = 15) and transgender men, treatment ongoing (*n* = 32).

	Baseline	12 months	Δ Estimated differences (95% CI)	*p*‐Value
Heart rate (bpm) TransM_TN TransM_TO	60 (55;65) 62 (56;66)	62 (57;68) 64 (60;68)	2.0 (−1;5) 2.0 (−1;6)	0.30 0.35
Intraventricular septum (cm) TransM_TN TransM_TO	0.6 (0.6;0.7) 0.7 (0.6;0.8)	0.7 (0.6;0.8) 0.8 (0.7;0.9)	0.08 (0.00;−0.16) 0.07 (0.00;−0.13)	0.05 0.06
Left ventricular end‐diastolic internal diameter (cm) TransM_TN TransM_TO	4.4 (4.2;4.6) 4.6 (4.3;5.0)*	4.7 (4.1;4.9) 4.6 (4.4;4.8)	0.1 (−0.1;0.3) −0.0 (−0.2;0.1)	0.20 0.60
Left ventricular end‐systolic internal diameter (cm) TransM_TN TransM_TO	2.9 (2.7;3.4) 3.2 (2.9;3.5)	3.2 (2.7;3.8) 3.1 (2.9;3.6)	0.4 (−0.1;0.8) −0.0 (−0.2;0.1)	0.12 0.65
Left ventricular mass index (g/m^2^) TransM_TN TransM_TO	60 (45;65) 62 (51;73)	68 (54;77) 66 (46;86)	6 (−11;22) 4 (−1;16)	0.47 0.09
Posterior wall diameter (cm) TransM_TN TransM_TO	0.8 (0.7;0.9) 0.8 (0.7;0.9)	0.7 (0.6;0.9) 0.8 (0.7;0.9)	−0.07 (−0.19;0.05) −0.03 (−0.10;0.05)	0.21 0.46
Left atrial volume index (mL/m^2^) TransM_TN TransM_TO	18 (15;22) 20 (17;24)	15 (13;22) 17 (13;23)	−3 (−8;1) −2 (−5;1)	0.11 0.156
Relative wall thickness TransM_TN TransM_TO	0.36 (0.33;0.40) 0.38 (0.34;0.41)	0.38 (0.34;0.42) 0.39 (0.35;0.43)	0.02 (−0.01;0.04) 0.01 (−0.01;0.03)	0.12 0.17
Biplane left ventricular ejection fraction (%) TransM_TN TransM_TO	57.0 (52.4;61.2) 56.5 (54.7;61.2)	57.9 (53.5;64.4) 57.1 (54.0;63.0)	1.5 (−4.1;7.2) 0.0 (−3.0;3.1)	0.57 0.98
Auto left ventricular ejection fraction (%) TransM_TN TransM_TO	56.0 (52.0;63.0) 56.0 (52.0;59.0)	57.0 (53.0;62.0) 56.0 (54.0;59.0)	0.5 (−4.2;5.2) 1.0 (−1.3;3.2)	0.82 0.39
Left ventricular global longitudinal strain (%) TransM_TN TransM_TO	−17.8 (−18.8;−16.5) −18.8 (−20.3;−17)	−17.4 (−19.2;−16.8) −18.2 (−19.9;−16.7)	−0.2 (−0.8;0.4) 0.1 (−0.6;0.7)	0.50 0.83
Stroke volume (ml) TransM_TN TransM_TO	64 (55;71) 63 (56;70)	66 (58;72) 64 (57;70)	2 (−2;7) 1 (−3;5)	0.19 0.47
Cardiac output (L/min) TransM_TN TransM_TO	4.8 (4.2;5.6) 5.0 (4.3;5.5)	5.0 (4.4;5.7) 5.1 (4.5;5.8)	0.2 (−0.6;0.8) 0.1 (−0.6;0.7)	0.15 0.54
Mitral E‐wave velocity (m/s) TransM_TN TransM_TO	0.82 (0.69;0.89) 0.81 (0.66;0.86)	0.89 (0.78;0.94) 0.78 (0.66;0.92)	0.08 (−0.02;0.18) 0.02 (−0.03;0.06)	0.10 0.44
Early left ventricular relaxation (septal) E’ (cm/s) TransM_TN TransM_TO	14 (12;18) 12 (11;14)	11 (10;15) 12 (11;14)	**−2 (−5;−2)** 0 (−5;6)	**0.04** 0.99
Early left ventricular relaxation (lateral) E’ (cm/s) TransM_TN TransM_TO	18 (16;22) 16 (12;20)	15 (13;17) 15 (12;17)	**−3 (−5;−1)** 0 (−6;6)	**0.02** 0.90

**Legend** Table 2: Baseline and 12 months data.

Data presented as median (interquartile range). Delta (Δ) differences present changes between baseline measurements and after one year of testosterone therapy for biochemical markers with normally distributed delta values. *P*‐values: paired comparison of the difference between baseline and 12 months.

Abbreviation: TransM_TN: Transgender men treatment‐naive, TransM_TO: Transgender men treatment ongoing.

*p < 0.05 when comparing values between new transgender males and transgender males already on treatment at baseline or 12 months, respectively.

### 3.4 Baseline compared to 1‐year assessments

In the TransM_TN group, septal and lateral left early ventricular relaxation decreased significantly (14–11 cm/s, *p* = 0.04 and 18–15 cm/s, *p* = 0.02, respectively. Septal and lateral left ventricular relaxation remained unchanged in the TransM_TO group.

A trend towards an increase in hemodynamic parameters, including stroke volume and cardiac output, was observed in both groups. LVEF and global longitudinal strain did not show significant changes in either group (Table 2).

No significant changes in left ventricular geometry were observed in either the TransM_TN or TransM_TO groups during follow‐up. However, the number of individuals with concentric remodeling increased from 1 to 2 in the TransM_TN group and from 1 to 4 in the TransM_TO group. The prevalence of concentric hypertrophy remained unchanged at 0 in the TransM_TN group but increased from 1 to 2 in the TransM_TO group. Data not shown.

**FIGURE 2:**
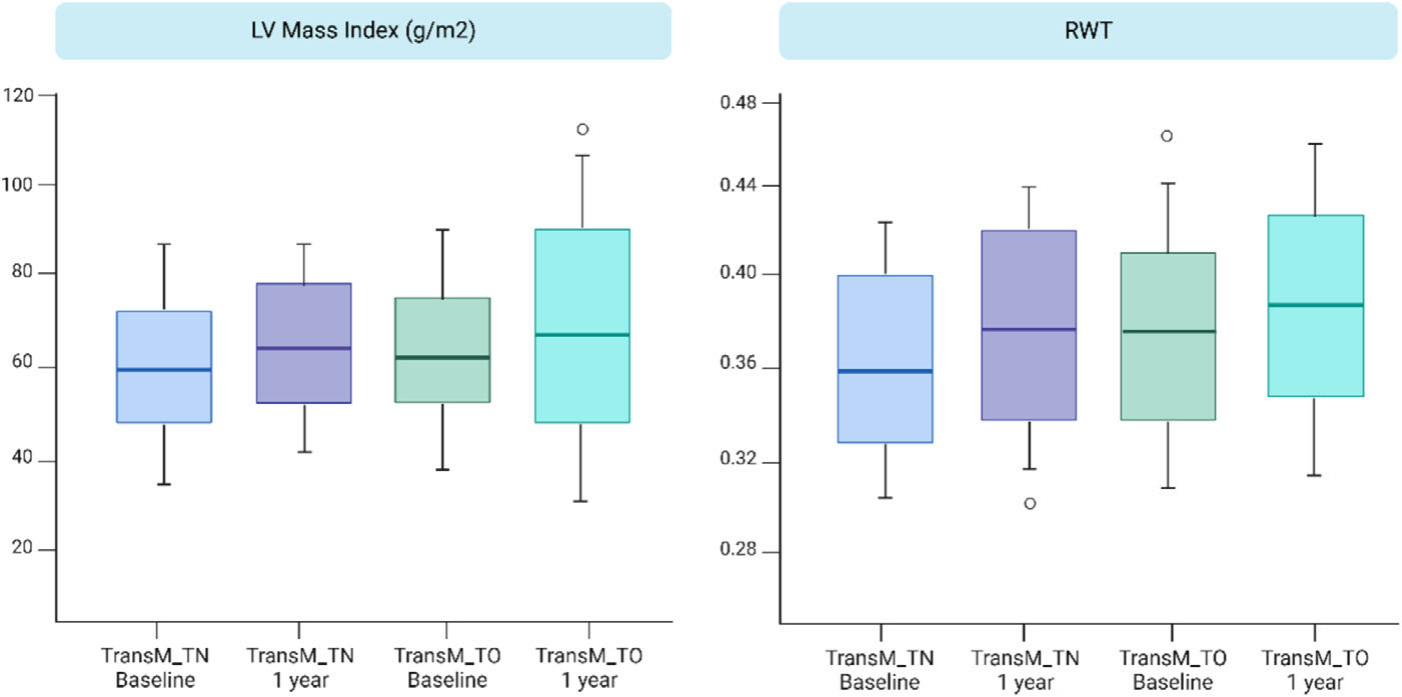
Changes in left ventricular (LV) Mass Index and relative wall thickness (RWT). Boxplots depicting the distribution of LV Mass Index (g/m^2^) and RWT among transgender men who are treatment‐naive (TransM_TN) and those undergoing treatment (TransM_TO). Data are shown at baseline and after 12 months.

**FIGURE 3:**
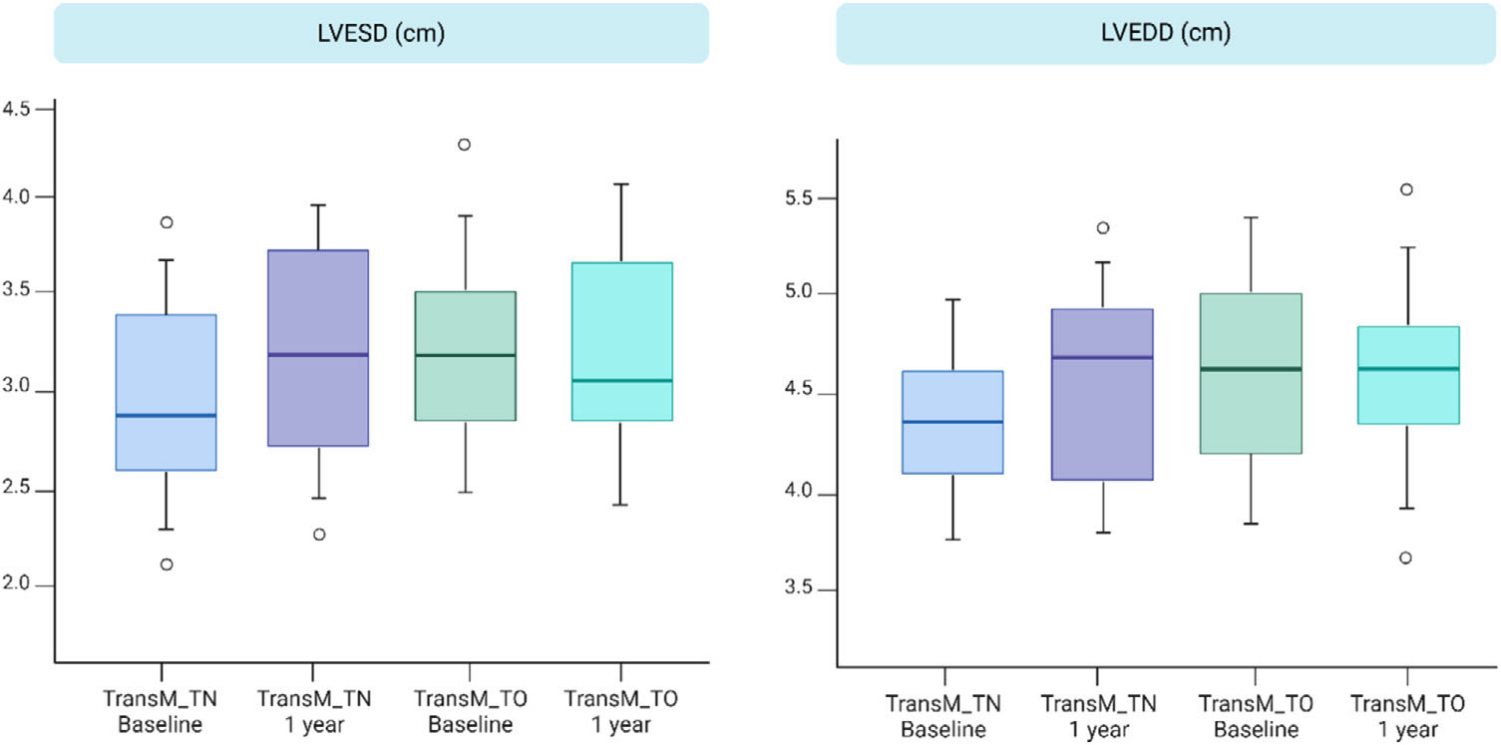
Changes in LVESD and LVEDD. Boxplots illustrating the echocardiographic measurements of transgender men, comparing those who are treatment‐naive (TransM_TN) and those undergoing treatment (TransM_TO), both at baseline and after 12 months of treatment. The parameters presented are LV end systolic diameter (LVESD, cm) and LV end diastolic diameter (LVEDD, cm).

During follow‐up, there was a trend towards increased left ventricular mass index and relative wall thickness in both groups (Figure 3), although these trends did not achieve statistical significance. Additionally, both Left ventricular end‐diastolic diameter and left ventricular end‐systolic diameter exhibited a tendency to increase from baseline to 12 months among TransM_TN, though these changes were not statistically significant (Figure 2).

Sensitivity analysis excluding participants with preexisting cardiovascular comorbidities, yielded results consistent with the original analysis. No significant changes in cardiac morphology, or systolic and diastolic function were identified, confirming that the presence of participants with preventive treatments did not significantly affect the overall findings.

### 3.5 Baseline assessments—TransM_TO versus TransM_TN

Left ventricular end‐diastolic diameter was significantly larger in the TransM_TO group at baseline compared to the TransM_TN group 4.6 cm (interquartile range [IQR] 4.3–5.0 cm) and 4.4 cm (IQR 4.2–4.6 cm) respectively (*p* < 0.05). Other morphological, systolic, and diastolic outcomes at baseline were comparable in the TransM_TN and TransM_TO groups.

### 3.6 Coronary CT angiography

CAC scores, non‐calcified plaque formation, or grade of coronary stenosis were unchanged after 1 year in the TransM_TN and TransM_TO groups.

CAC score increased in two participants in the TransM_TO group. One non‐smoker, aged > 40 years with no prior cardiovascular disease, familial coronary disease history, or hypercholesterolemia, the baseline CAC score was 0 at baseline and 2 after one year of GAHT. The second participant was a non‐smoker, age > 50 years diagnosed with diabetes, and hypertension, but normal cholesterol and no family history of coronary disease, the CAC score increased from 54 to 68 after one year. CAC scores, non‐calcified plaque formation, or grade of coronary stenosis were not significantly different at baseline in the TransM_TN group compared to the TransM_TO group.

## 4 DISCUSSION

The present study aimed to address the impact of masculinizing GAHT on key cardiovascular health outcomes over the course of one year in both treatment‐naive transgender males and those already undergoing GAHT.

We observed a decline in left ventricular relaxation in TransM_TN initiating GAHT, with significant reductions in septal and lateral early relaxation velocities, indicating a possible decline in diastolic function. There were also indications of increased left ventricular volume after one year, though not statistically significant. At baseline, TransM_TO individuals on GAHT for a median duration of 4 years had significantly larger left ventricular end‐diastolic diameters compared to TransM_TN, suggesting the potential for structural changes in cardiac dimensions over time with ongoing masculinizing hormone therapy.

Changes in diastolic function during masculinizing GAHT may either reflect direct effects of GAHT on myocardial structure and function ^38^ or indirect effects of GAHT on hemodynamic conditions, body composition, or fluid volume. For instance, a small study demonstrated that a saline infusion causes changes in mitral annular velocities.^39^ In the present study, masculinizing GAHT was followed by minor decreases in transmitral flow (early ventricular filling velocities) and increases in left ventricular cavity size, which are often associated with an increase in fluid load.^40^ Additionally, in a study involving men with growth hormone (GH) deficiency and hypogonadism, testosterone was found to independently increase extracellular fluid volume.^41^ Therefore, our findings suggest that the observed decrease in septal and lateral relaxation velocities could be a consequence of increased volume load. A 3 cm/s reduction in early ventricular relaxation velocity indicates mild diastolic dysfunction. While statistically significant, this change is unlikely to have immediate clinical implications. However, persistent diastolic dysfunction could lead to more pronounced cardiac remodeling over time. Similarly, a 0.2 cm increase in left ventricular diameter may represent early structural adaptation rather than pathological changes but requires further observation. Additional prospective studies are essential to better understand these findings.

We observed no signs of deteriorating left ventricular function over the 1‐year follow‐up period. This was indicated by stable LVEF and global longitudinal strain, along with no significant changes in left ventricular geometry (concentric remodeling and/or hypertrophy). Furthermore, at baseline, no significant differences were observed in almost all echocardiographic variables between individuals before initiating GAHT and those who had been masculinizing GAHT for several years (median of 4 years). This consistency in cardiac measurements between TM_TN and TM_TO supports the overall short‐term cardiovascular safety of GAHT. Furthermore, the sensitivity analysis confirmed the robustness of our findings, demonstrating that the observed results were not influenced by participants with baseline cardiovascular risk conditions. This strengthens our conclusions, highlighting that the reported trends reflect group‐level changes rather than individual variations linked to comorbidities or inherited predispositions.

By including participants regardless of their pretreatment risk profile, we were able to explore whether adverse cardiovascular outcomes might be more prevalent in individuals with a more unfavorable pretreatment risk profile.

Left ventricular geometry was evaluated using a left ventricular mass index threshold of 95 g/m^2^, which corresponds to the cut‐off value for cisgender women. This choice of threshold is particularly intriguing and warrants further discussion regarding whether transgender men should be evaluated using male or female reference values. We found no statistically significant increase in left ventricular mass, however, we observed a trend toward higher left ventricular mass index and relative wall thickness in transgender men after initiating GAHT. This trend suggests that individuals using masculinizing GAHT will develop cardiac characteristics more typical of cisgender men rather than cisgender women. In accordance, a recent paper demonstrated that trans‐gender women shared more left ventricular geometry characteristics with matched cis‐gender women than cis‐gender men.^42^

Regarding indicators of coronary atherosclerosis, we found no significant changes in CAC scores, non‐calcified coronary plaque, or coronary stenosis grade between baseline and 1‐year measurements. Notably, the participant group in the present study comprised young individuals with a low baseline risk for atherosclerosis. The only observed changes in CAC scores occurred in two older individuals, likely influenced by factors such as age, hypertension, and lifestyle factors like smoking or diabetes, making it difficult to attribute the changes solely to GAHT. While these findings suggest that GAHT may not increase the short‐term risk of atherosclerotic disease in transgender males, the small sample size remains a significant limitation. Additionally, we observed a notable decrease in HDL‐C levels, which aligns with findings from previous studies documenting similar changes in lipid profiles associated with GAHT.^11^, ^12^, ^13^ Although we observed no significant development of atherosclerosis in the short term, persistently altered lipid profiles during GAHT may lead to coronary plaque formation and increased CVD risk over time.^43^

This study has several limitations that should be considered. First, the small sample size of 47 participants, divided into two subgroups (treatment‐naïve and treatment‐ongoing), limits the statistical power to detect subtle or less frequent cardiovascular changes. While we observed significant echocardiographic changes after 1 year of masculinizing GAHT, these findings highlight the need for long‐term follow‐up studies. Reproduction of our results in larger, multicenter studies is crucial to confirm and extend these observations. A 1‐year follow‐up period may be insufficient to capture long‐term cardiovascular effects, particularly those associated with progressive atherosclerosis or sustained cardiac remodeling. Changes such as coronary plaque development or clinically significant cardiac dysfunction often require extended observation to become evident. To address this limitation, the ongoing study has extended the follow‐up interval for coronary CT angiography to five years, which is expected to provide more comprehensive insights into long‐term cardiovascular outcomes. The study was designed as a paired analysis, with transgender men serving as their own controls, which is a notable strength. However, the cohort was relatively young, and the follow‐up duration was limited to one year. While prospective data from untreated transgender men are challenging to obtain, future studies in aging cohorts should include cisgender controls of the same and opposite birth sex.

Additionally, the majority of participants used IM testosterone injections, which may have influenced the observed outcomes. However, the study lacked sufficient power to stratify the cohort by treatment modality (transdermal vs. IM injections), limiting the ability to explore modality‐specific effects.

Despite these limitations, this study provides valuable insights into the potential mechanisms underlying adverse cardiovascular outcomes in transgender men. Large‐scale national, multicenter, and registry‐based studies are crucial for understanding the long‐term impact of GAHT on clinical cardiovascular endpoints, such as myocardial infarction, heart failure, and cardiovascular mortality. Nevertheless, our approach enabled the assessment of short‐term effects of GAHT from initiation through ongoing treatment, laying a foundation for future research and follow‐up studies.

In conclusion, we observed a decline in diastolic function in transgender men initiating masculinizing GAHT. Although no deterioration in overall left ventricular function was identified, ongoing GAHT was associated with larger left ventricular volumes compared to transgender persons before initiating GAHT. No significant atherosclerotic progression was observed during the short‐term course of masculinizing GAHT.

## AUTHOR CONTRIBUTIONS

Laust Frisenberg Buhl led the study's investigation, data collection, formal analysis, and manuscript drafting as the corresponding author. Louise Lehmann Christensen, Marianne S. Andersen, Jan Frystyk, and Dorte Glintborg provided supervision, secured funding, and contributed to conceptualization and review. Axel Diederichsen, Selma Hasific, Jordi Sanchez Dahl, Kirstine Nørregaard Hansen, and Manijeh Noori assisted with cardiovascular data collection. Rikke Hjortebjerg contributed to statistical analysis, while Gitte Maria Jørgensen supported radiology data collection. Camilla Viola Palm and Tine Taulbjerg Kristensen assisted with overall data management. All authors reviewed and approved the final manuscript.

## CONFLICT OF INTEREST STATEMENT

The authors declare no conflict of interest.

## ETHICS STATEMENT

All participants provided informed consent. The study was conducted in accordance with the guidelines of the Declaration of Helsinki and received ethical approval from the Ethics Committee of the Region of Southern Denmark (protocol code S‐20210078).

## CLINICAL TRIAL REGISTRATION

The trial was registered on ClinicalTrials.gov (Identifier: NCT04254354)

## Data Availability

The data that support the findings of this study are available on request from the corresponding author. The data are not publicly available due to privacy or ethical restrictions.
